# A New Approach for the Production of Selenium-Enriched and Probiotic Yeast Biomass from Agro-Industrial by-Products in a Stirred-Tank Bioreactor

**DOI:** 10.3390/metabo10120508

**Published:** 2020-12-13

**Authors:** Sabrina Evelin Martiniano, Letícia Alves Fernandes, Edith Mier Alba, Rafael Rodrigues Philippini, Stephanie Caroline Tavares Tabuchi, Marek Kieliszek, Júlio César dos Santos, Silvio Silvério da Silva

**Affiliations:** 1 Engineering School of Lorena, University of São Paulo, Lorena 12.602-810, SP, Brazil; leticia_fernandes_27@hotmail.com (L.A.F.); edithmier@usp.br (E.M.A.); philippinir@gmail.com (R.R.P.); stephanietabuchi@gmail.com (S.C.T.T.); jsant200@usp.br (J.C.d.S.); silviosilverio@usp.br (S.S.d.S.); 2 Department of Food Biotechnology and Microbiology, Institute of Food Sciences, Warsaw University of Life Sciences—SGGW, 02-776 Warsaw, Poland; marek_kieliszek@sggw.edu.pl

**Keywords:** selenium, selenium-enriched, *Saccharomyces cerevisiae*, probiotic yeast, hydrolysate, agro-industrial wastes, corn bran, soybean bran, bioreactor

## Abstract

The production of biomolecules using agro-industrial by-products as feedstock is a growing trend worldwide. Selenium (Se) is a trace element essential for health, and the Se-enrichment of yeast biomass can enhance its benefits. This study investigated the feasibility of the production of *Saccharomyces cerevisiae* Se-enriched biomass using a medium composed of corn bran and soybean bran acid hydrolysates as carbon and nitrogen sources in a stirred-tank reactor. After hydrolysis, hydrolysates presented complex composition and high concentrations of sugars, proteins, and minerals. The use of a stirred-tank bioreactor leads to the production of 9 g/L *S. cerevisiae* biomass enriched with 236.93 μg/g Se, and 99% cell viability. Likewise, the combination of sugarcane molasses and soybean bran hydrolysate was effective for cell growth of a probiotic strain of *S. cerevisiae* with a 24.08% β-glucan content. The results demonstrated that starchy acid hydrolysates are low-cost and efficient substrates for the production of yeast biomass and derivate products and may contribute to further studies for a sustainable development of biorefinery technologies.

## 1. Introduction

The consumption of yeast biomass brings several health benefits. Nutritional yeasts can be divided into probiotics and prebiotics (metabolites isolated from biomass) [1]. Probiotic yeasts belong to a group of living microorganisms whose consumption in adequate amounts confers health benefits [2]. In animals, they balance the gut microbiota, improve dry matter digestibility [3], and assist in health maintenance. Some desirable characteristics for probiotic yeasts are tolerance to pH and bile salts, hydrophobicity, auto-aggregation, antioxidant, and biosecurity properties [4]. These abilities ensure the capacity to grow inside the digestive tract and the gut microbiome, and the control of pathogens [4,5].

The use of yeasts as prebiotics includes inactivated microorganisms, fragments from the cell wall, autolyzed yeasts, and mineral-enriched biomass [1]. β-glucan is a major component in the fungal cell wall and plays an important role in health due to its immunomodulatory and antitumor properties [6]. The dry weight of *Saccharomyces cerevisiae* cell wall consists of 30–60% of β-glucan, 1–2% chitin, and different proteins linked to glycans [7]. Still, yeasts can be enriched with minerals to increase their nutraceutical properties.

Selenium (Se) has an important role for human and animal health due to its antioxidant and antitumor properties, protection against drug-induced toxicity, regulation of thyroid gland function, prevention of inflammatory diseases, and also acting in animal growth and performance [8,9,10,11]. Low levels of Se have been associated with cardiovascular diseases and immune dysfunction [10]. This element has a narrow range between its health requirement and toxicity that vary among animal species, and it might cause toxic effects in organisms when supplemented in high concentrations by increasing oxidative stress [12].

In nature, Se is found under different oxidation states (2^−^, 2^+^, 4^+^, 6^+^, and “zero”, a biologically inert form) and it has a critical role as the redox center in selenoproteins such as glutathione peroxidase (GPx), thioredoxin reductase (TrxR), and selenoprotein P [13]. Se is present in soil and water and enters the food chain through plants, which uptake it from the soil [14]. However, it is found in low concentrations in the environment and the biofortification of food and feed is a common method to overcome health problems.

Animals can consume Se in both organic and inorganic form, even though organic Se has a higher bioavailability for organisms [11,15]. Furthermore, the organic form of Se-enriched yeast can enhance the vaccine conferred immune response against the influenza virus in chicken [16]. Due to its importance in animal performance, most Se enrichment studies have been focused on the development of this mineral tolerance by microorganisms, plants, and small animals commonly used as feed such as yeasts [17], edible fungi [18], olive leaves [19], earthworms [20], rotifers [21], shrimps [22]. Among these groups, yeasts are well-known sources of organic Se for animals and play an important role in the industry as feed/food components and diet supplements.

Yeasts can tolerate various Se concentrations and require simple media composition for growth. Besides, whether compared to plants, these microorganisms can incorporate more Se due to their high protein content [23]. Yeasts can also bind Se in both inorganic and organic forms through extracellular and intracellular bioaccumulation, respectively [9]. The intracellular bioaccumulation occurs via the sulfur pathway for amino acid biosynthesis [23,24].

The global single-cell protein market is expected to present a compound annual growth rate of 18.1% during the forecast period of 2016–2022 and was estimated at USD 12.7 billion in 2016 [25]. The Se-enriched yeast production generally uses feedstocks rich in free sugars such as glucose [26], sugarcane molasses [27], grape juice [28], malt extract [29], sprouted brown rice, soybean sprout juices [29]. These types of substrates do not require complex pretreatment but might compete with food production. The use of agro-industrial by-products as feedstock is a low-cost alternative for the biotechnological production of Se-enriched yeast [30], and assists in the biorefinery implementation, especially for developing countries. The bioresource management is essential for the fulfillment of the needs of humankind and has an important role in the economy; besides, secondary bioresources such as the agro-industrial by-products do not directly compete with food production and enable the use of agricultural lands for other purposes [31].

Bran is an agro-industrial by-product from grain manufacturing and, though it is partially used as feed, a large amount remains as a residue. This biomass is rich in macronutrients and micronutrients and can be used as both carbon and nitrogen source for microorganisms during the production of several bioproducts such as Se-enriched yeast, single-cell protein, biopigments, β-glucans, ethanol, xylitol [30,31,32,33]. Agro-industrial by-products composition may vary depending on the region, however, the basic constituents of each by-product remain in the same range [33,34]. A biomass pretreatment is necessary to release sugars and nutrients before fermentation, and the acid pretreatment is a low-cost and efficient method for bran such as corn bran [35]. Besides, the use of agro-industrial by-products for the production of mineral-enriched yeast biomass can be integrated into biorefineries platforms, promoting the simultaneous development of an inexpensive mineral-enriched protein source [31]. Thereby, their use as carbon and nitrogen sources for Se yeast enrichment is a recent and eco-friendly technology that assists in the development of biorefineries. The present study aims to evaluate the production of Se-enriched yeast biomass and probiotic yeast biomass in a stirred-tank reactor using a medium composed of agro-industrial by-products as carbon and nitrogen sources.

## 2. Results and Discussion

### 2.1. Starchy Hydrolysates Characterization

Before pretreatment, corn bran and soybean bran presented 9.06% and 8.36% water content, respectively. The acid hydrolysis of corn bran released more sugars (175.72 ± 19.97 g/L total sugars, and 47.01 ± 0.85 g/L reducing sugars) than the acid hydrolysis of soybean bran (38.61 ± 4.45 g/L total sugars, and 12.48 ± 0.62 g/L reducing sugars) (Table 1). The major sugar release in corn bran can be related to biomass composition since starch corresponds about to 32–49% of this biomass [35,36].

Xylose, arabinose, and phenolic compounds have been found in both CBAH (corn bran acid hydrolysate) and SBAH (soybean bran acid hydrolysate). The presence of pentose sugars is due to the hemicellulosic fraction in brans [37] and phenolic compounds are possibly from the lignin fraction since this macromolecule is found in some starchy by-products composition [38] and, besides, brans may contain husks from the manufacturing process. Furthermore, hemicellulose corresponds to 15–35% of plant structure and it is mainly composed of pentoses such as xylose [39]. Lee et al. [35] reported xylose as the main released sugar, followed by glucose and arabinose, in destarched corn bran hydrolysate after enzymatic pretreatment using cellulolytic and hemicellulolytic enzymes. Sanchez et al. [38] reported the presence of hemicellulosic sugars and lignin in rice bran. Probst and Vadlani [40] found 25.8% hemicellulose and 1.4% lignin in corn bran, being xylose the major sugar in hemicellulosic fraction.

SBAH presented higher protein concentration (13.52 ± 1.06 g/L) than CBAH (7.82 ± 2.33 g/L), which can be related to the high protein content reported for this biomass. Barzegar et al. [36] reported 47.5% protein in soybean meal and 8.6% protein in corn. Corn bran has a lower protein concentration than soybean, Sousa et al. [41] reported a range of 8.08–12.39% protein, and Probst and Vadlani [40] found 11.6% protein in corn bran.

Regarding mineral composition, SBAH demonstrates a higher concentration of macrominerals and microminerals than CBAH, as can be observed in Table 1. In general, soybean has higher mineral content than corn, i.e., Reddy et al. [42] found macronutrients concentrations P, K, Ca, and Mg up to 14.1 g/kg in soybean seeds, while Sousa et al. [41] detected maximum levels up to 8.7 g/kg in corn bran for these same minerals.

### 2.2. Fermentation in Stirred Tank Bioreactor (STR) for Selenium Enrichment

The medium presented 44.23 ± 5.48 g/L reducing sugars and 11.42 ± 2.44 g/L total protein before the fermentation assays. At 72 h, cells were recovered by centrifugation and the selenium content was determined. *S. cerevisiae* 193 presented 9.0 ± 0.26 g/L cell biomass (d.w., dry weight) enriched with 236.93 μg/g Se and 99 ± 1% viability at 72 h. Until 24 h cultivation, *S. cerevisiae* 193 presented rapid cell growth, but the growth remained reasonably stable posteriorly, and cell biomass exhibited a pink-red color starting at 24 h. The color change is probably due to Se biotransformation inside cells since a red color in yeast biomass indicates the reduction of selenite or selenate into elemental Se [9,25].

Most studies of Se enrichment in microorganisms utilize glucose as a carbon source. In the study of Zhang et al. [43], the authors produced 13.32 g/L of *C. utilis* biomass enriched with 1010 μg/g Se after 18 h fermentation using YPD medium added by 15 mg/L Na_2_SeO_3_. Likewise, Kieliszek et al. [44] produced 14.1 g/L of *C. utilis* biomass with 1841 μg/g Se content after 48 h using a YPD medium added by 30 mg/L Se as Na_2_SeO_3_ in a stirred tank bioreactor. Yang et al. [18] obtained 905.2 and 984.7 μg/g Se content in *C. utilis* biomass in medium supplemented with 15 mg/L Na_2_SeO_3_ under batch and fed-batch systems, respectively. In another study, Kieliszek et al. [45] reported a Se content of 1800 μg/g in 15 g _d.w._/L of *C. utilis* biomass and 800 μg/g in 6.7 g _d.w._/L of *S. cerevisiae* biomass after 72 h cultivation. Using agro-industrial by-products as substrate, Martiniano et al. [31] obtained 4.25 g/L biomass containing 167 μg/g Se from corn bran acid hydrolysate supplemented with 15 mg/L Se as Na_2_SeO_3_, and Egressy-Molnár [46] obtained 42.3 μg/g Se content in *Hericium erinaceus* mushroom biomass using substrate composed of sawdust, wooden chips, and wheat bran.

The maximum cell biomass yield (Y_X/S_) occurred at 48 h (1.77 g/g), but the major volumetric cell productivity (Q_X_) was observed at 24 h (0.25 g/L h) (Table 2).

At 72 h, *S. cerevisiae* 193 metabolized 11.46% reducing sugars of medium and the protein concentration did not suffer any significant alteration (11.58 ± 2.85 g/L). The low reducing sugar and protein consumption may be indicative that the yeast utilized other sources for growth, and, likewise, the fermentation time may be decreased since the maximum biomass yield and volumetric cell productivity occurred before 72 h.

The presence of a high concentration of Se has an inhibitory effect in yeast growth [43,47], but 10 mg Se^4+^/L did not inhibit the growth of the evaluated strain. The higher Se concentration in the medium leads to lower cell growth, but different species exhibit different ranges of tolerance towards this compound [31,48]. Furthermore, when the medium contains nonfermentable carbon sources, cells are more oxygen-demanded, and free radicals are generated during respiration, which can cause oxidative stress to cells, reducing cell growth and viability [24]. In the study of Zhang et al. [43], the authors obtained *Candida utilis* biomass yield of 0.45 g/g using medium supplemented with 15 mg/L Se. Santos et al. [49] produced *Candida utilis* biomass using sugarcane vinasse as substrate and obtained 2.25 g/L biomass, 0.13 g/g yield, and 0.08 g/L h productivity. Juszczyk et al. [50] studied the biomass production of *Yarrowia lipolytica* yeast using raw glycerol from flaxseed oil production, resulting in a biomass yield of 0.51 g/g and productivity of 1.33 g/L h.

Some *S. cerevisiae* strains can colonize oily residues such as wastewater from table olive processing [51], and both CBAH and SBAH presented oil content, which could be visually observed during the hydrolysate treatment, possibly due to the starchy biomass composition. Sousa et al. [41] reported 7.69–13.85% lipid content in corn bran and Hartman et al. [52] reported 18% lipid in soybean seeds. Besides, starchy biomass has a complex composition and it is composed of a wide range of mono- and oligosaccharides, starch, fibers, sucrose, fatty acids, protein, minerals, ashes, etc. [36,38,40,41,42,52,53]. Therefore, hydrolysates from agro-industrial by-products are complex media and may contain complex carbohydrates and other carbon sources, as well as a wide range of mineral content. Consequently, the medium presented pentose sugars, acetic acid, phenolic compounds, and sugar degradation products (5-hydroxymethylfurfural and furfural), which might act as inhibitors for yeast growth.

Neither ethanol nor glycerol production was observed during the fermentation, possibly due to aeration conditions and strain characteristics. Although glycerol and ethanol are common by-products in fermentative processes using yeasts, their production is not always desirable since it is associated with the redox balance in yeasts and can decrease the yeast biomass production due to the deviation of growth metabolism [31,54]. However, the presence of Se in the medium can also lead to a major bioethanol production in some yeast strains as a response to stress conditions [31,48] and it might assist in further studies for the integration of mineral-enrichment, single-cell protein, and biofuel production in biorefineries.

Even though the Se-enrichment of yeasts has been studied over the years, the use of agro-industrial wastes as feedstock brings a new option especially for developing countries such as Brazil, one of the main producers of protein sources with large area occupied by agriculture. The use of crop by-products represents an important technological advance since most technologies are focused on the development of integrated crop-livestock systems to optimize the yields [55]. Thus, crop wastes are produced in large amounts due to the improvement of agriculture activities and represent important sources of nutrients that can be used for microbial growth [33]. *S. cerevisiae* is an important microorganism used for bioethanol production due to its ability to ferment sugars and its high ethanol productivity and tolerance [56]. This yeast enrichment with Se leads to a new possibility of integration in biorefineries approaches, as a by-product, since yeast from distilleries is usually offered as protein for animal nutrition [57].

### 2.3. Evaluation of Biomass Production and β-Glucan Content of Probiotic Yeast in Stirred-Tank Bioreactor

**Figure 1:**
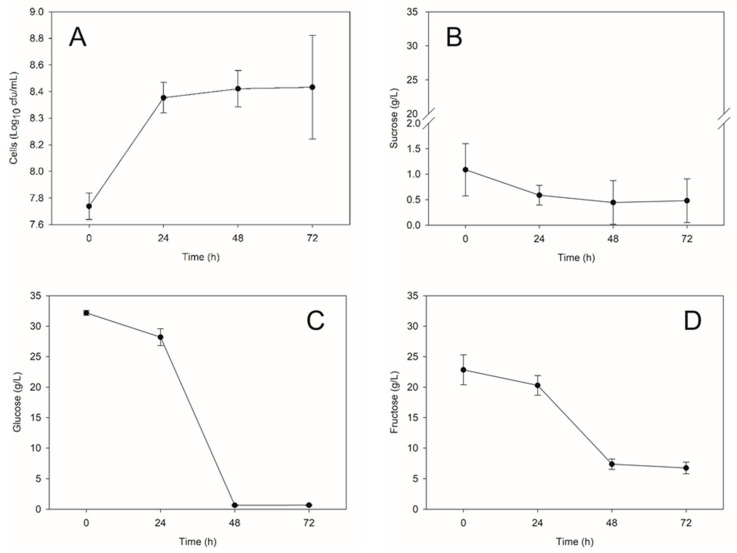
Cell growth (**A**) and the consumption of sucrose (**B**), glucose (**C**), and fructose (**D**) by the probiotic yeast *Saccharomyces cerevisiae* Sc47 in a medium composed of sugarcane molasses and soybean bran acid hydrolysate.

The medium presented an initial sugars concentration of 32.01 ± 0.44 g/L glucose, 22.9 ± 2.5 g/L fructose, and 1.1 ± 0.5 g/L sucrose. At 72 h, the production of cell biomass was 6.6 ± 1.1 g/L with 95± 2% viability. Glucose was rapidly depleted by 48 h, followed by the consumption of fructose and sucrose (Figure 1). However, fructose was not fully consumed during the evaluated time of cultivation, which may be related to a preferential transport of glucose over fructose into the cell. The maximum volumetric productivity (Q_X_) equaled 0.1 g/L h and occurred at 48 h, with yields (Y_X/S_) of 0.152 and 0.311 g/g in glucose and sucrose, respectively. At 48 h, the yeast consumed 98% of glucose, 68% of fructose, and 59% of sucrose from the medium. After this time, the concentration of sugars remained stable. It is indicative that the time of the fermentation process could be shorted, reducing the costs of the process.

The low biomass production might be related to the temperature chosen for fermentation, 37 °C since the yeast capacity to grow at a temperature tolerated by probiotic yeasts was evaluated [58]. Although this strain was able to grow at 37 °C and some *Saccharomyces cerevisiae* strains are thermotolerant, this species is a mesophilic microorganism [59]. Thereby, the temperature might have interfered with cell growth. 

Cangussu et al. [3] produced 2.5 g/L, Y_X/S_ of 0.02 g/g, and Q_X_ of 0.21 g/L h of a probiotic yeast in an STR bioreactor using YPD medium under microaerobic conditions at 40 °C and 150 rpm. Guluarte et al. [4] obtained about 4.0 × 10^6^ of a probiotic *Kluyveromyces lactis* using YPD medium with pH 5.5 and incubated at 29 °C. Although the use of synthetic and semisynthetic media is a well-established method for cell growth, the agro-industrial by-products are low-cost alternatives for the production of yeast biomass. Paula et al. [60] produced a probiotic wheat beer using *S. cerevisiae* var. *boulardii* as brewer’s yeast. These authors observed that glucose was preferably assimilated in the wheat beer wort, followed by maltose, and the strain performance was sensitive over temperature. Singu et al. [61] used a thermo-tolerant *Saccharomyces cerevisiae* var. *boulardii* strain coated with hydrocolloids to produce probiotic cornflakes.

Sugarcane molasses is a by-product with a complex composition and rich in sugars, which correspond up to 50% of molasses composition, primarily consisting of glucose, sucrose, and fructose [62]. Likewise, the soybean bran acid hydrolysate presented a complex composition. Agro-industrial by-products can be utilized as a nutrient source for microbial growth, but it is important to have adequate medium composition and fermentation parameters since these biomasses might present some natural variations in their composition.

Putra et al. [63] produced 10 g/L of *S. cerevisiae* biomass from date palm waste using an airflow of 1.25 vvm and pH range of 5.0–5.6. Santos et al. [49] obtained 2.3 g/L of *Candida utilis* biomass from vinasse, a residue generated after cachaça (special alcohol from Brazil) and ethanol production. In a study using molasses, Vu et al. [64] produced 36.5 g/L of *S. cerevisiae* using molasses and corn steep liquor under optimized conditions in an STR bioreactor.

At 72 h, cells were recovery by centrifugation and treated for the extraction of β-glucan from the cell wall. The recovered β-glucan corresponded to 24.08 ± 2.14% from the disrupted yeast biomass. In general, β-glucan comprises 15–30% of the dry weight of the yeast cell [6]. However, the efficiency of β-glucan recovery varies according to the extraction methods [65]. Hong et al. [66] observed 9.42% of β-glucan content in a probiotic *S. cerevisiae,* whereas Pengkumsri et al. [67] obtained up to 41.69% and Kim and Yun [68] recovered 13% from nonprobiotic *S. cerevisiae* strains.

The yeast *S. cerevisiae* is a widely recognized source of fungal β-glucan [6]. Yeasts are commonly present as components in animal feed [34] and bring several health benefits due to their probiotic and prebiotic properties. β-glucan from the fungal cell wall improves the animal performance and, when conciliated with the probiotic properties of some yeast strains, might increase the health potential of yeast biomass altogether.

## 3. Materials and Methods

### 3.1. Obtention and Pretreatment of Agro-Industrial by-Products

**Figure 2:**
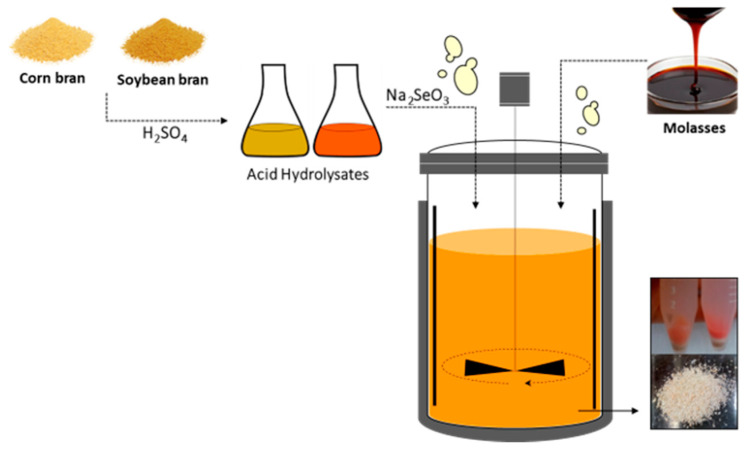
Scheme of preparation media of the cultivation yeast.

Corn bran, soybean bran, and sugarcane molasses (Caseiro and Natural, Brazil) were acquired from the local market (Lorena, SP, Brazil). The moisture contents of corn bran and soybean bran were determined by infrared radiation at 105 °C in analytical balance. Corn bran hydrolysis was carried out in stainless steel reactor of 80 L operating volume filled with 40 L solution using 1% H_2_SO_4_ (*w*/*v*), 1:8 solid/liquid ratio (*w*/*v*) (dry mass) at 121 °C for 15 min. Soybean bran was hydrolysate with H_2_SO_4_ 1% (*w*/*v*), 1:5 (*w*/*v*) (dry mass) in Erlenmeyer flasks in an autoclave at 121 °C, 15 min. After hydrolysis, both hydrolysates were filtered in cotton cloth, had their pH adjusted with 6.5 mol/L NaOH until pH 5.5, were centrifuged (2000× *g*, 10 min), sterilized (1.1 atm, 15 min), and centrifuged aseptically in these same conditions [33]. Sugarcane molasses was diluted until 50 g/L of reducing sugars, the pH was adjusted to 5.5 using 6.5 mol/L NaOH, and sterilized at 1.1 atm for 15 min (Figure 2).

### 3.2. Characterization of Starchy Acid Hydrolysates and Sugarcane Molasses

Starchy hydrolysates in natura were characterized in triplicate for reducing sugars by DNS (2,5-dinitrosalicylic acid) method [68], total proteins [69], mineral content by inductively coupled plasma optical emission spectrometry (ICP-OES) (Perkin Elmer-Optima 8000), and pH. The presence of glucose, xylose, arabinose, and cellobiose in starchy hydrolysates and molasses were determined by high-performance liquid chromatography (HPLC) in Agilent Technology chromatograph equipped with an aminex HPX-87H (BIORAD) column (300 × 7.8 mm) at 45 °C, using 0.005 mol/L H_2_SO_4_ solution as eluent, with a flow rate of 0.6 mL/min, refractive index detection (RID6A), and injection of 20 μL of samples. Sugarcane molasses was also characterized regarding pH, reducing sugars (DNS method), and the concentration of sucrose and fructose by HPLC under the conditions of HPX-87H (BIORAD) column (300 × 7.8 mm) at 60 °C, deionized water as eluent, and 0.4 mL/min flow rate.

### 3.3. Microorganisms and Inoculum

*Saccharomyces cerevisiae* 193 (CRM-UNESP–Rio Claro, SP, Brazil) and a commercial probiotic strain *S. cerevisiae* Sc 47 (Actisaf^®^—Lesaffre, Campinas, Brazil), were used in this investigation. Yeasts were grown in YPMG agar composed of (g/L) glucose 10.0, peptone 5.0, malt extract 3.0, yeast extract 3.0, and agar 20.0. The incubation of *S. cerevisiae* 193 and *S. cerevisiae* Sc 47 agar plates occurred at 30 °C for 48 h.

The inoculum was prepared by the transference of two loopful of colonies of each strain from YPMG agar to YPG broth containing (g/L) glucose 30.0, peptone 20.0, and yeast extract 10.0. Erlenmeyer flasks were filled with 40% working volume with YPG broth and incubated in an orbital shaker at 30 °C, 200 rpm for 24 h [33]. After 24 h, the inoculum was centrifuged at 2000× *g* for 10 min for cell recovery, and the yeast biomass was washed, suspended in sterile distilled water, and adjusted for 1 × 10^8^ cell/mL by counting cells in Agasse Lafont-R chamber (0.0025 mm^2^ × 0.1 mm^3^) (Optik Labor, Lancing, United Kingdom) under 400× magnification in an optical microscope [31].

### 3.4. Production of Se-Enriched Yeast Biomass in a Stirred-Tank Bioreactor

The medium CSBAH was composed of corn bran acid hydrolysate (CBAH) and soybean bran acid hydrolysate (SBAH) at a 4:1 (*v*/*v*) ratio, containing about 40 g/L of reducing sugars and 10 g/L total protein, 10 mg/L of Se as Na_2_SeO_3_, and initial pH of 5.5.

Fermentation assays were performed in triplicate in a stirred-tank reactor (KLF2000—Bioengineering, Switzerland) with 1.5 L capacity and 1 L working volume, equipped with sensors of pH and temperature, four removable baffles, an agitator shaft with two standard six-bladed impellers, and a glass condenser. Fermentation conditions were 30 °C, 350 rpm, 1.5 vvm [70] for 72 h, taking samples every 24 h for the determination of cell growth, cell viability, substrate consumption, and by-products production (ethanol and glycerol). At the end of the process, cells were collected by centrifugation (2000× *g*, 10 min), washed with distilled water, and stored at 4 °C for the analysis of total Se.

### 3.5. Production of Probiotic Yeast Biomass in a Stirred-Tank Bioreactor

The evaluation of cell biomass production and β-glucan content of the probiotic yeast *S. cerevisiae* Sc 47 was performed in triplicate in a stirred-tank bioreactor equipped according to Section 3.6. The medium was composed of the agro-industrial by-products of sugarcane molasses and SBAH in a ratio of 4:1 (*v*/*v*). Sugarcane molasses was diluted until 40 g/L of reducing sugars, and the initial pH was adjusted to 5.5 using 6.5 mol/L NaOH. Fermentation was carried out at 37 °C, 350 rpm, 1.5 vvm for 72 h [58,70]. The cultivation was performed using a commercial probiotic strain without the addition of Se to evaluate the potential use of agro-industrial by-products hydrolysates for the growth of probiotic yeasts. Likewise, fermentation assays were carried out under 37 °C according to Porto et al. [58] for probiotic yeasts performance evaluation.

### 3.6. Fermentation Analytical Methods and Kinetic Parameters

Cell growth was determined in triplicate by counting cells on the Agasse Lafont-R chamber (0.0025 mm^2^ × 0.1 mm^3^) (Optik Labor, Lancing, UK) at 400× in an optical microscope [71]. Besides, the cell growth was also determined by optical density at 600 nm, correlated with a previously performed growth curve (Equation (1)) as well. Cell viability was determined by mixing the diluted cell suspension with a 0.01% methylene blue solution (1:1, *v*/*v*).

The cell biomass yield (Y_X/S_, g/g) was calculated by the relation between the concentration of cell biomass (dry weight) produced by the concentration of reducing sugars consumed. The volumetric productivity (Q_X_, g/L h) was calculated using the biomass production versus time.

(1) y = 2.2042x + 0.0974

Ethanol, xylitol, glycerol, glucose, xylose, arabinose, and cellobiose were analyzed in triplicate by HPLC in Agilent Technology chromatograph (aminex HPX-87H (Bio-Rad)) column—300 × 7.8 mm, 45 °C, 0.005 mol/L H_2_SO_4_ as eluent, 0.6 mL/min flow rate, refractive index detection RID6A, and injection of 20 μL of samples. Reducing sugars were determined by the 2,5-dinitrosalicylic acid (DNS) method [68] and total proteins were determined by the Lowry protein assay [69].

### 3.7. Determination of Selenium Uptake in the Se-Enriched Yeast Biomass

For Se analysis, *S. cerevisiae* 193 biomass was previously suspended in deionized water and digested with H_2_SO_4_ (1:1.5, *v*/*v*) in Erlenmeyer flasks covered with glass funnel, and maintained at 300 °C for 1 h in a heating plate, followed by the addition of HNO_3_ 1:1 (*v*/*v*), and heating at 270 °C for 2 h [70]. The solutions of digested cells were cooled at room temperature and transferred to volumetric flasks for Se determination by ICP-OES (Perkin Elmer, Optima 8000) at 196.026 nm using Na_2_SO_3_ (1000 mg/L) as standard [31]. All assays were performed in triplicate.

### 3.8. Determination of β-Glucan in Probiotic Yeast Biomass

In this first approach, β-glucan content was evaluated only in *S. cerevisiae* Sc47 biomass aiming to conciliate both probiotic and prebiotic properties in a single product. All assays were performed in triplicate. Yeast was disrupted using autolysis. In this process, a suspension of 45 g/L cell biomass in distilled water was incubated at 50 °C, 120 rpm, for 48 h, and subsequently, at 80 °C for 15 min [65]. After this process, the cell biomass was recovered by centrifugation at 2000× *g* for 10 min and the supernatant was stored at 4 °C.

The extraction of β-glucan from the disrupted cell biomass was carried out following a method adapted from Kim and Yun [67]. In this method, cells were treated with 2% NaOH (*w*/*v*) and incubated in a water bath at 90 °C, for 5 h, centrifuged at 2000× *g* for 10 min, and neutralized until pH 7.7 with 2 mol/L HCl. Ethanol at −10 °C was added to the cell suspension in a ratio of 3:1 (*v/v*) and the mixture was left at 4 °C for 24 h. The β-glucan precipitate was oven-dried at 60 °C until constant and determined gravimetrically.

### 3.9. Statistical Analysis

The data obtained from this study were subjected to analysis of variance using Statistica 13.3 program (StatSoft Inc., Tulsa, OK, USA). The significance of differences between the mean values in each group was tested by Tukey’s test at a significance level of *α* = 0.05.

## 4. Conclusions

The use of corn bran and soybean bran acid hydrolysates as substrate enabled the production of 9.0 g/L *S. cerevisiae* 193 biomass enriched with 236.93 μg/g Se after 72 h cultivation in an STR bioreactor. A medium elaborated with sugarcane molasses and soybean bran hydrolysate is efficient for cell growth of a probiotic strain of *S. cerevisiae*. Yet, further studies of fermentation parameters are necessary to up-scale these processes. Agro-industrial by-products are efficient substrates for the biotechnological production of yeast biomass and derivate products that can be produced altogether with biofuels for a sustainable development of biorefinery technologies.

## Figures and Tables

**Table 1:** Concentration of sugars, proteins, acetic acid, phenolic compounds, and sugar degradation products in corn bran, and soybean bran starchy acid hydrolysates.

Composition	Corn Bran Hydrolysate	Soybean Bran Hydrolysate
	(g/L)	
Total sugars	175.72 ± 19.97 ^a,^*	38.61 ± 4.45 ^b^
Reducing sugars	47.01 ± 0.85 ^b^	12.48 ± 0.62 ^c,d^
Glucose	4.83 ± 1.2 ^c,d,e^	2.2 ± 1.16 ^d,e^
Xylose	3.69 ± 0.66 ^c,d,e^	4.41 ± 1.41 ^c,d,e^
Arabinose	3.35 ± 0.51 ^c,d,e^	0.44 ± 0.02 ^e^
Cellobiose	5.35 ± 0.28 ^c,d,e^	2.56 ± 0.27 ^d,e^
Proteins	7.82 ± 2.33 ^c,d,e^	13.52 ± 1.06 ^c^
Phenols	0.39 ± 0.05 ^e^	0.33 ± 0.05 ^e^
Acetic acid	0.33 ± 0.26 ^e^	0.42 ± 0.01 ^e^
Glycerol	0.67 ± 0.39 ^e^	1.69 ± 0.48 ^d,e^
	**(mg/L)**	
5-HMF	NA	101 ± 13
Furfural	NA	10 ± 7
Fe	5.14 ± 0.90 ^b,^*	9.88 ± 8.94 ^b^
Ca	18.94 ± 8.89 ^b^	149.91 ± 56.9 ^b^
Mg	111.98 ± 29.66 ^b^	486.35 ± 167.41 ^b^
Mn	NA	1.19 ± 1.96 ^b^
K	463.38 ± 77.96 ^b^	4908.25 ± 1423.76 ^a^
Na	NA	10.90 ± 11.63 ^b^
Se	0.05 ± 0.04 ^b^	0.33 ± 0.11 ^b^

NA = not available, 5-HMF = 5-(Hydroxymethyl)furfural. *^,a–e^Means with the same letter did not differ significantly (acc. Tukey’s HSD test).

**Table 2:** Kinetic parameters of cell biomass yield (Y_X/S_), volumetric cell productivity (QX), and substrate consumption (Y_C_) by *Saccharomyces cerevisiae* 193 cultivated in a medium composed of corn bran and soybean bran acid hydrolysates enriched with 10 mg/L selenium as Na_2_SeO_3_.

Parameter	Time		
	24 h	48	72
Y_X/S_ (g/g)	1.76 ± 0.28	1.77 ± 0.17	1.29 ± 0.07
Q_X_ (g/L.h)	0.25 ± 0.04	0.12 ± 0.01	0.09 ± 0.005
Y_C_ (%)	8.22 ± 0.26	5.50 ± 1.14	11.46 ± 0.65
